# Circulating biomarkers of kidney angiomyolipoma and cysts in tuberous sclerosis complex patients

**DOI:** 10.1016/j.isci.2024.110265

**Published:** 2024-06-13

**Authors:** Varvara I. Rubtsova, Yujin Chun, Joohwan Kim, Cuauhtemoc B. Ramirez, Sunhee Jung, Wonsuk Choi, Miranda E. Kelly, Miranda L. Lopez, Elizabeth Cassidy, Gabrielle Rushing, Dean J. Aguiar, Wei Ling Lau, Rebecca S. Ahdoot, Moyra Smith, Aimee L. Edinger, Sang-Guk Lee, Cholsoon Jang, Gina Lee

**Affiliations:** 1 Department of Biological Chemistry, School of Medicine, University of California Irvine, Irvine, CA, USA; 2 Department of Microbiology and Molecular Genetics, School of Medicine, University of California Irvine, Irvine, CA, USA; 3 TSC Alliance, Silver Spring, MD, USA; 4 Division of Nephrology, Department of Medicine, School of Medicine, University of California Irvine, Irvine, CA, USA; 5 Division of Genetics and Metabolism, Department of Pediatrics, School of Medicine, University of California Irvine, Irvine, CA, USA; 6 Department of Developmental and Cell Biology, School of Biological Sciences, University of California Irvine, Irvine, CA, USA; 7 Department of Pharmaceutical Sciences, School of Pharmacy and Pharmaceutical Sciences, University of California Irvine, Irvine, CA, USA; 8 Chao Family Comprehensive Cancer Center, University of California Irvine, Irvine, CA, USA; 9 Department of Laboratory Medicine, Yonsei University College of Medicine, Seoul, Korea; 10 Center for Complex Biological Systems, University of California Irvine, Irvine, CA, USA; 11 Center for Epigenetics and Metabolism, University of California Irvine, Irvine, CA, USA

**Keywords:** Clinical genetics, Endocrinology, Pathophysiology

## Abstract

Patients with tuberous sclerosis complex (TSC) develop multi-organ disease manifestations, with kidney angiomyolipomas (AML) and cysts being one of the most common and deadly. Early and regular AML/cyst detection and monitoring are vital to lower TSC patient morbidity and mortality. However, the current standard of care involves imaging-based methods that are not designed for rapid screening, posing challenges for early detection. To identify potential diagnostic screening biomarkers of AML/cysts, we performed global untargeted metabolomics in blood samples from 283 kidney AML/cyst-positive or -negative TSC patients using mass spectrometry. We identified 7 highly sensitive chemical features, including octanoic acid, that predict kidney AML/cysts in TSC patients. Patients with elevated octanoic acid have lower levels of very long-chain fatty acids (VLCFAs), suggesting that dysregulated peroxisome activity leads to overproduction of octanoic acid via VLCFA oxidation. These data highlight AML/cysts blood biomarkers for TSC patients and offers valuable metabolic insights into the disease.

## Highlights

• Untargeted metabolomics screen in a large cohort of TSC patients’ blood
• 40 circulating chemical features elevated in TSC patients with kidney AML/cysts
• 7 representative features, including octanoic acid, mark AML/cyst in TSC
• High octanoic acid is associated with low VLCFA, suggesting activated peroxisomes

## Graphical abstract

**Figure:**
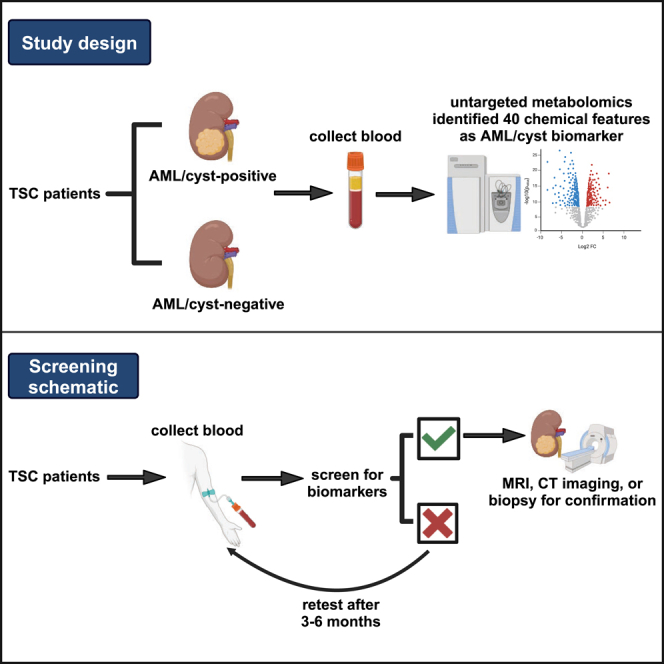

## Introduction

Tuberous sclerosis complex (TSC) is an autosomal dominant disease that produces neurological manifestations and benign tumors in multiple organs, including the brain, heart, lung, skin, and kidneys.^1^^,^^2^^,^^3^ TSC occurs in about every 1 in 6,000 births, and afflicts about 2 million people worldwide.^4^^,^^5^^,^^6^ TSC most commonly results from *de novo* spontaneous mutations in *TSC1* or *TSC2* tumor suppressor genes but can also be inherited from parents.^7^^,^^8^

Among TSC-associated manifestations, kidney angiomyolipomas (AML), which are benign tumors composed of blood vessels, muscle, and fat, are the leading cause of death in TSC patients.^9^^,^^10^ AML/cysts develop in 80% of TSC patients during childhood and persistently progress throughout their lives.^11^^,^^12^^,^^13^ Treatment of AML/cysts becomes necessary when they become large (>3 cm diameter) and pose a risk for life-threatening rupture and hemorrhage.^1^^,^^13^^,^^14^^,^^15^ Therefore, appropriate lifelong surveillance and management of AML/cysts are crucial. Abdominal imaging is recommended at the time of TSC diagnosis since kidney AML can be asymptomatic prior to substantial growth.^16^^,^^17^ Magnetic resonance imaging (MRI) is the preferred modality because it can detect even fat-poor AML with higher accuracy than abdominal ultrasonography or computed tomography (CT). However, definitive diagnosis of AML requires biopsy, which is highly invasive.^18^ Further, annual surveillance imaging^17^ can pose a financial burden for many patients. There is thus an unmet need for blood biomarkers that can non-invasively and cost-effectively indicate the presence of kidney AML/cysts that require additional treatment. To address these issues and improve the care of AML/cysts in TSC patients, developing an efficient and accessible method of diagnosis is necessary. Such biomarkers obtained through simple blood testing would allow physicians to screen for the presence of AML/cysts before performing confirmatory imaging and biopsy-based diagnostics.

Studies have identified blood-borne proteins as biomarkers that can distinguish TSC patients from healthy controls as well as indicate the development of neurological manifestations.^19^^,^^20^^,^^21^ To date, however, studies linking metabolomic changes to TSC renal manifestations are limited in scope.^22^ The use of modern mass spectrometry-based untargeted metabolomics has expanded the discovery of circulating disease biomarkers.^23^ Using global untargeted metabolomics in 283 TSC patients with or without AML/cysts, here we report 7 potential blood biomarkers. In particular, the accumulation of octanoic acid in AML/cyst-positive TSC patients provides insight into TSC-specific metabolic alterations such as overactive peroxisome-mediated fatty acid oxidation.

## Results

### Demographic and clinical characteristics of the study population

We obtained blood samples from 283 patients with TSC confirmed by clinical diagnostic criteria alone (65 patients) or genetic testing (218 patients) through the TSC Alliance Biosample Repository and Natural History Database^24^ (Table S1). Patients were divided into two groups, kidney AML/cyst-positive (*n* = 232) or -negative (*n* = 51), based on imaging results from CT, MRI, and ultrasonography. Population characteristics including age, sex, and *TSC* mutation, are shown in Table 1. The majority of AML/cyst-positive patients had a *TSC2* mutation (51.7%), with only 10.8% having a *TSC1* mutation. This high rate of *TSC2* mutation was consistent with other patient cohorts.^13^^,^^25^^,^^26^ Only five patients had mutations in both *TSC2* and *PKD1* genes, a condition that causes both TSC and autosomal dominant polycystic kidney disease (ADPKD).^27^ Among these patients, all five had renal cysts, and three of them also had kidney AML (Table S1). Among the 120 patients with multiple AML lesions, 111 patients had bilateral AML. Other common TSC-related manifestations, including brain subependymal giant cell astrocytoma (SEGA) and cardiac rhabdomyoma, exhibited similar prevalence in the two groups. Out of 283 patients, 117 patients had been treated with mechanistic target of rapamycin (mTOR) inhibitors at the time of blood sampling with a median treatment duration of 1743.5 days (Table S2). The use of mTOR inhibitors was more prevalent in AML/cyst-positive (45.3%) than -negative (23.5%) patients (Table 1).

**Table 1:** Demographic and clinical characteristics of the study population

	TSC with kidney AML or Cysts (*N* = 232)	TSC with Normal Kidney (*N* = 51)	*p* value
Age in years, median (range)	16.1 (0.9–58.0)	13.3 (0.8–52.9)	0.1015

**Gender, n (%)**			

Women	123 (53.0)	23 (45.1)	0.3055
Men	109 (47.0)	28 (54.9)	

**TSC mutation, n (%)**			

TSC1 mutation	25 (10.8)	13 (25.5)	0.0059
TSC2 mutation	120 (51.7)	15 (39.4)	
TSC1&2 mutations	8 (3.4)	4 (7.9)	
No	19 (8.2)	7 (13.7)	
Unknown	60 (25.9)	12 (23.5)	

**SEGA, n (%)**			

Yes	66 (28.4)	9 (17.7)	0.1395
No	155 (66.8)	37 (72.5)	
Unknown	11 (4.8)	5 (9.8)	

**Cardiac Rhabdomyoma, n (%)**			

Yes	120 (51.7)	25 (49.0)	0.9224
No	93 (40.1)	22 (43.1)	
Unknown	19 (8.2)	4 (7.9)	

**mTOR inhibitor use, n (%)**			

Yes	105 (45.3)	12 (23.5)	0.0043
No	127 (54.7)	39 (76.5)	

**Renal manifestation, n (%)**			

Cysts	90 (38.8)		
AML	32 (13.8)		
AML and Cysts	110 (47.4)		
Bilateral AML, n (%)	112/142 (78.9)		
Multiple AML lesions, n (%)	120/142 (84.5)		
AML >3 cm, n (%)	44/142 (31.0)		

### Select chemical features elevated in AML/cyst-positive TSC patients

**Figure 1:**
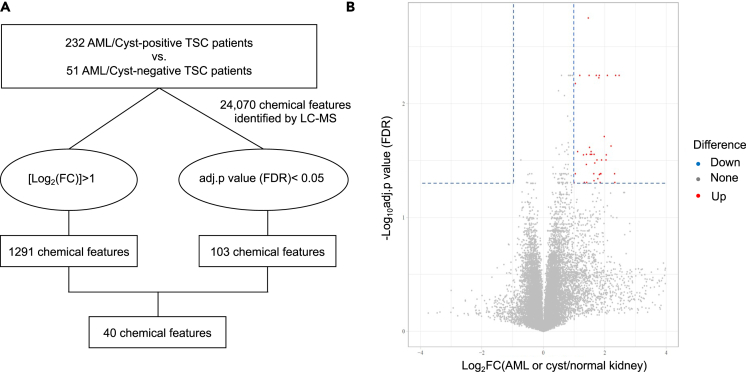
Strategy for metabolomics studies to find plasma chemical features associated with kidney AML/cyst in TSC patients (A) Overall schematic to find plasma chemical features associated with kidney AML/cyst. (B) Volcano plot showing 40 chemical features (red dots) applying selection criteria, a |log2(fold change)|>1 and an adjusted *p* value<0.05 by Student’s t test followed by FDR correction. *N* = 232 and 51 for AML/cyst-positive and -negative TSC patients.

To identify potential blood biomarkers of AML/cyst-positive patients, we performed untargeted metabolomics using high-resolution, high-sensitivity liquid chromatography-mass spectrometry (LC-MS). This comprehensive screen yielded 24,070 distinct chemical features, including many unknowns (Figures 1A and 1B). We selected potential biomarkers by applying selection criteria of a **|**log_2_(fold change)**|** > 1 and an adjusted *p* < 0.05 after false-discovery rate (FDR) correction. A total of 40 chemical features passed these thresholds (Table 2). For some of these features, we were able to assign chemical formulas and putative metabolites using accurate mass-to-charge (m/z) values matching publicly available chemical libraries (see STAR Methods). We validated one of the chemical features, m/z = 144.11485, as octanoic acid based on m/z and retention time matching with the authenticated chemical standard (Figure S1).

**Table 2:** Characteristics and subgroups of 40 chemical features monitored by LC-MS

Group	MW	RT [min]	Presumed Formula	Presumed metabolite	*p* value	Adjusted *p* value	log_2_FC	Max peak area
1	144.11485	2.568	C8 H16 O2	Octanoic acid	0.0001	0.042	1.823	3,629,164,488
1	145.11829	2.637			<0.0001	0.024	2.203	227,283,883
1	142.09936	2.494	C8 H14 O2	2-Octenoic acid	<0.0001	0.006	2.085	135,327,156
1	140.08387	2.56	C8 H12 O2	(4Z,6Z)-octa-4,6-dienoic acid	<0.0001	0.028	2.049	70,468,118
1	160.10987	3.696	C8 H16 O3	Hydroxyoctanoic acid	<0.0001	0.019	1.984	59,543,089
1	316.116	3.706	C13 H21 N2 O5 P		0.0001	0.041	2.319	20,759,655
1	144.05988	2.609			<0.0001	0.006	2.466	15,804,941
1	226.11805	2.566	C8 H14 N6 O2		0.0001	0.046	1.775	13,716,502
1	146.12139	2.568	C8 H19 P		0.0001	0.041	1.874	11,591,925
1	318.13144	3.703	C13 H23 N2 O5 P		0.0001	0.031	2.040	11,469,040
1	144.16983	2.708			0.0002	0.049	2.328	10,854,572
1	308.19859	2.602	C19 H24 N4		<0.0001	0.028	1.527	5,164,287
1	310.21191	2.574	C18 H30 O4	13(S)-HpOTrE isomer	0.0001	0.031	1.746	4,615,159
1	318.13129	2.823	C12 H25 N4 P3		<0.0001	0.006	2.353	3,183,375
1	251.11909	2.21	C10 H21 N O4 S		0.0001	0.033	1.674	3,129,135
1	317.12632	2.814	C18 H15 N5 O		0.0002	0.049	1.850	2,583,266
1	519.36766	2.563	C31 H54 N O P S		0.0001	0.041	1.871	2,165,917
1	72.02022	2.542			<0.0001	0.026	1.555	1,948,348
1	352.09259	3.707	C13 H26 N2 O P2 S2		<0.0001	0.028	1.653	1,862,380
1	319.13508	3.691			<0.0001	0.028	1.574	1,489,247
1	354.10813	3.688	C14 H27 O4 P S2		<0.0001	0.024	1.505	1,150,452
1	196.0865	3.704	C7 H17 O4 P		<0.0001	0.006	1.807	1,134,695
1	144.13386	2.568			0.0001	0.031	1.895	1,042,501
1	224.10234	2.5	C8 H12 N6 O2	2,6-Diamino-9-(2-hydroxyethoxymethyl)purine	0.0001	0.034	1.400	950,238
1	272.17364	3.676	C13 H24 N2 O4	N-octanoylglutamine	<0.0001	0.028	1.405	933,994
1	188.0781	2.613	C7 H12 N2 O4	N-acetyl-glutamine	<0.0001	0.006	1.723	749,064
1	493.09786	2.611	C19 H28 N O8 P S2		0.0001	0.041	1.040	654,803
1	142.15304	2.496			<0.0001	0.006	1.822	432,102
1	344.72821	2.545			<0.0001	0.026	1.116	368,752
1	517.3518	2.496	C25 H51 N5 O2 S2		<0.0001	0.006	1.185	217,261
1	144.10583	2.494	C8 H17 P		<0.0001	0.006	1.491	195,330
2	211.08192	13.339	C5 H13 N3 O6		0.0001	0.041	1.856	711,451
2	249.0285	13.339	C4 H9 N7 O2 P2		0.0002	0.048	1.649	476,283
2	268.0729	12.913	C8 H18 N2 O4 P2		0.0001	0.041	1.642	422,898
2	351.0831	12.049	C15 H18 N3 O3 P S		0.0002	0.049	1.414	255,705
3	315.24	2.972			0.0002	0.049	1.001	11,839,214
4	233.06623	1.885	C6 H12 N5 O3 P		<0.0001	0.007	1.041	4,754,931
5	386.05722	2.6	C12 H15 N6 O5 P S		0.0002	0.049	1.335	2,705,805
6	194.04241	3.707	C7 H14 O2 S2		<0.0001	0.028	1.307	1,927,228
7	470.23385	2.454	C19 H40 N2 O7 P2		<0.0001	0.002	1.464	1,320,292

**Figure 2:**
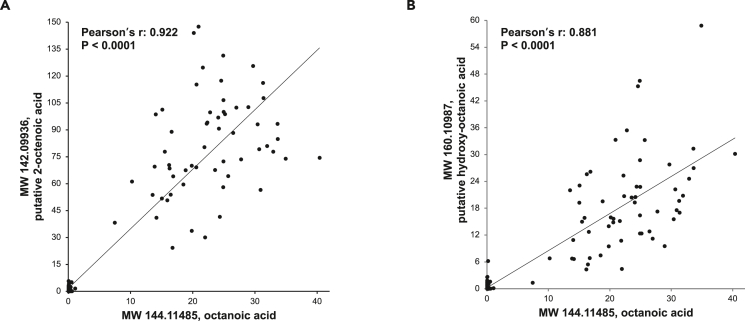
Correlation analysis between the identified chemical features (A) Correlation between MW 144.11485 (octanoic acid) and MW 142.09936 (putative 2-octenoic acid). (B) Correlation between MW 144.11485 (octanoic acid) and MW 160.10987 (putative hydroxy-octanoic acid). *P* values by Pearson’s chi-squared test. *N* = 232 AML/cyst-positive TSC patients.

We next performed a systematic correlation analysis to understand the biochemical relationships between our identified chemical features. Interestingly, we found significant positive correlations between several chemical features (Figures S2 and S3). Most correlations were observed between chemical features that have different retention times, suggesting that they are distinct metabolites in related biochemical pathways. For example, octanoic acid exhibited strong positive correlations with putative 2-octenoic acid (Pearson’s r = 0.922) and hydroxy octanoic acid (Pearson’s r = 0.881) (Figures 2A and 2B). Based on our systematic correlation analysis, we separated the chemical features into 7 groups (Table 2). The chemical features within each group highly correlated with each other, with a correlation coefficient (r) greater than 0.5 and a *p* value of less than 0.001. Using this analysis, we sorted 31 chemical features into group 1 (Figure S2) and 4 features into group 2 (Figure S3).

**Figure 3:**
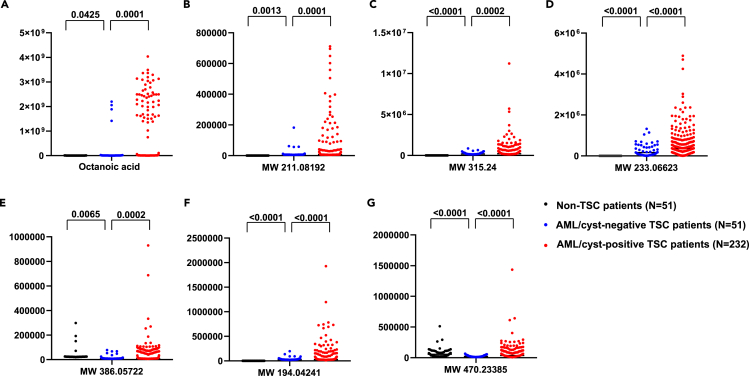
Comparison of abundance of the 7 selected chemical features between plasma samples from non-TSC, AML/cyst-negative TSC, and AML/cyst-positive TSC patients (A–G) Y axis indicates ion counts. Note that the abundance of these chemical features is significantly higher in the AML/cyst-positive TSC patients than the other groups, with octanoic acid in (A) showing the greatest differences. Numbers indicate *p* values by Student’s t test. *N* = 51, 51, and 232 patients for each group.

We next obtained 51 non-TSC patients’ blood to examine whether our identified chemical features are specific to AML/cyst-positive TSC patients (Figures 3A–3G). Basic characteristics of these non-TSC patients are summarized in Table S3, and any patients diagnosed with kidney or metabolic diseases (e.g., obesity, diabetes) were excluded. For this analysis, we selected the chemical feature with the highest peak area in groups 1 and 2, as well as the individual chemical features that made up groups 3 to 7 (Table 2). Comparison of the abundance of the 7 chemical features between the three patient groups (non-TSC vs. AML/cyst-negative TSC vs. -positive TSC) showed significantly higher levels of these chemicals in AML/cyst-positive TSC patients than the other two groups, with octanoic acid level showing the biggest difference (Figure 3A).

### Combining biomarkers improves clinical performance in detecting AML/cysts

**Figure 4:**
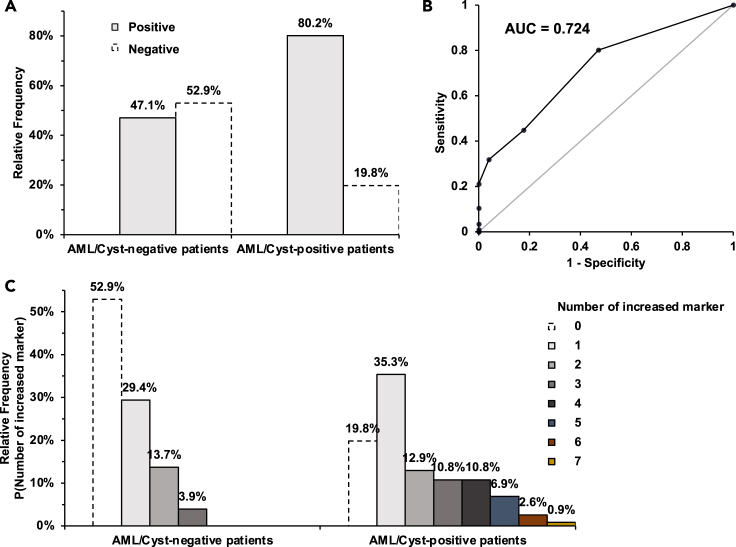
Clinical performance of blood test combining 7 chemical features in detecting AML/cyst (A) Relative frequency of AML/cyst-negative vs. -positive patients that are either positive or negative on the test. Positive means one of the 7 chemical features is elevated above the cut-off. (B) Receiver operating characteristic (ROC) curve of the blood test. (C) Distribution of the number of positive markers in AML/cyst-negative vs. -positive patients. Note that no AML/cyst-negative patients showed >3 positive markers. In contrast, the majority of AML/cyst-positive patients showed at least 2 markers positive.

We then evaluated the collective clinical performance of these 7 chemical features. Following a typical approach to determining biomarker cut-offs,^28^ we considered a blood test positive if any single chemical feature is elevated above the cut-off, which is set at the 90^th^ percentile of AML/cyst-negative TSC patients (Table S4). Encouragingly, the frequency of positives (clinical sensitivity) for the diagnosis of AML/cysts according to the increase in one of the 7 chemical features was high (80.2%) (Figure 4A). We found AML/cyst-negative patients have a 47.1% frequency of false positives (Figure 4A). These false-positive patients may have small or fat-poor AML/cysts undetected by less sensitive ultrasonography. Also, for some patients, blood was collected several months after the latest imaging-based monitoring, raising the possibility that AML/cysts developed by the time of blood collection. Given that a circulating marker-based method is envisioned as an initial diagnostic screen to identify TSC patients with AML/cysts that would require confirmative imaging- and biopsy-based diagnosis, future studies expanding this panel are warranted to increase the true positive rate to nearly 100% while maintaining a tolerable false positive rate.

To gauge the sensitivity and specificity of disease biomarker combinations, a receiver operating characteristic (ROC) curve is widely used, with an area under the curve (AUC) above 0.7 being regarded as an acceptable diagnostic marker.^29^ Importantly, the combination of our 7 chemical features yielded an AUC of 0.724 (Figure 4B), indicating that they meet field standards for adequate diagnostic markers. Among the combination of 7 chemical features, designating positivity as the elevation of any single marker showed the most favorable clinical performance with the highest Youden’s index (Table S5). For AML/cyst-negative patients, 29.4% showed one marker positive but the percentile decreased dramatically with more markers, with no patients showing >3 positive markers (Figure 4C). In contrast, the majority of AML/cyst-positive patients showed at least 2 markers positive. This analysis supports the use of combined markers to enhance the sensitivity for AML/cyst detection.

### Elevated octanoic acid suggests activated peroxisome in some TSC patients

The identification of three elevated 8-carbon compounds (octanoic acid, putative 2-octenoic acid, and putative hydroxy octanoic acid) in a subpopulation of AML/cyst-positive TSC patients (Figure 2) motivated us to scrutinize the underlying biology, which may reflect AML/cyst formation and its influence on kidney metabolism. Firstly, we found that no population characteristics, including age, sex, or the presence of other organ disorders, correlated with the elevation of these 8-carbon compounds (not shown). Furthermore, neither *TSC* mutation type (*TSC1* or *TSC2* or both) nor mTOR inhibitor treatment explained the variable 8-carbon compound levels (Table S2). Nevertheless, as mTOR activity measurements were not conducted in parallel with blood sampling, we cannot rule out the possibility that the lack of correlation is due to variable mTOR activity among patients. Secondly, the rise of 8-carbon compounds could be due to variations in diet. For example, a fat-enriched ketogenic diet is frequently used to manage epilepsy in TSC patients.^4^ Indeed, high octanoic acid in blood persisted over time in almost all patients with serial blood samples (on average, 843 days apart) (Figure S4; Table S6), which is consistent with the idea that long-term dietary habits may drive their increased levels. However, according to the patient survey, only two patients had a ketogenic diet at the date of blood sampling, and one patient showed normal octanoic acid levels while the other patient showed high levels (Table S7). Moreover, we found neither medium nor long-chain fatty acids, the main components of ketogenic diet, to be elevated in patients with high octanoic acid (Figure S5). Also, beta-hydroxybutyrate, a ketone body, was not elevated in patients with high octanoic acid (Figure S6). Thus, the select increase of 8-carbon compounds but no other fatty acids or ketones argues against dietary fat intake driving the elevation in 8-carbon biomarkers.

**Figure 5:**
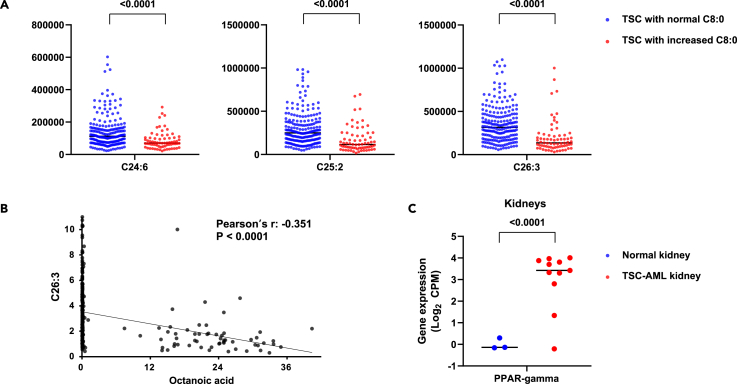
Blood octanoic acid increase is linked to peroxisome activity (A) Ion counts of plasma VLCFAs in AML/cyst-positive patients with normal vs. increased octanoic acid. Numbers indicate *p* values by Student’s t test. (B) A significant negative correlation between the abundance of octanoic acid and that of very long-chain fatty acid (VLCFA) C26:3 in AML/cyst-positive patients. *P* value by Pearson’s chi-squared test. (C) Gene expression of peroxisome proliferator-activated receptors gamma (PPAR-gamma) in normal kidney (*N* = 3) vs. TSC-AML kidney (*N* = 11). Numbers indicate *p* values by Student’s t test.

**Figure 6:**
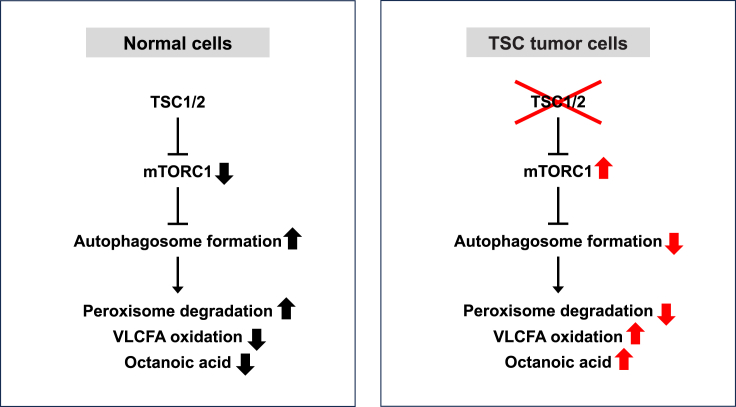
Schematic of increased octanoic acid and decreased very long-chain fatty acid (VLCFA) levels in AML/cyst-positive TSC patients In the peroxisomes, VLCFAs are oxidized to octanoic acid. In normal cells, the TSC1/2 was shown to degrade peroxisomes by autophagosome formation.^44^^,^^45^ In cells without TSC1/2 activity, mTORC1 is constitutively active and peroxisomes can remain intact and continue to oxidize VLCFAs, which leads to increased production of octanoic acid.

Aside from diet, octanoic acid can be generated endogenously by peroxisomes, an organelle where very long-chain fatty acids (VLCFAs) are oxidized into octanoic acid. Octanoic acid is then further oxidized in mitochondria.^30^^,^^31^ In this scenario, elevated octanoic acid may reflect highly active peroxisomal VLCFA oxidation, unmatched by subsequent mitochondrial oxidation. Indeed, patients with high octanoic acid levels had significantly lower VLCFAs in plasma (Figure 5A; Table 3), suggesting their accelerated breakdown. Further, there was a significant negative correlation between octanoic acid and many VLCFAs (Figure 5B; Table 3). RNA-sequencing data of kidney tissues from TSC and normal patients^32^ consistently shows that the kidneys from TSC patients with AML exhibit elevated expression of *peroxisome proliferator-activated receptors gamma* (*PPAR-gamma*), which may mediate VLCFA oxidation^33^ (Figure 5C). Together, these data suggest that elevated octanoic acid likely reflects hyperactive peroxisomal VLCFA oxidation in the kidneys of TSC patients with AML/cysts (Figure 6).

**Table 3:** Difference in free fatty acid levels between TSC patients with increased octanoic acid and those with normal octanoic acid

FFA	Mean of peak area		p	p.adj	log2FC	Pearson r (correlation with octanoic acid)	p (correlation)
	TSC patients with increased octanoic acid (*n* = 70)	TSC patients with normal octanoic acid (*n* = 213)					
C8:0, Octanoic acid	1,897,578,088	6,390,747	<0.0001	<0.0001	8.21396		
C14:1	11,891,687	14,213,042	0.1140	0.4430	−0.25726	−0.06615	0.2674
C14:2	2,550,417	3,221,597	0.0320	0.2018	−0.33704	−0.113	0.0575
C15:0	16,104,376	13,509,906	0.2570	0.6410	0.25344	0.08924	0.1343
C15:1	2,079,072	1,486,704	0.2580	0.6419	0.48382	0.1061	0.0748
C15:2	77,465	73,599	0.7820	0.9301	0.07385	0.01379	0.8174
C16:0, Palmitic acid	759,476,278	816,473,146	0.2040	0.5868	−0.1044	−0.0803	0.178
C16:2	2,138,223	2,424,823	0.1630	0.5321	−0.18147	−0.07694	0.1969
C17:1	9,234,860	9,662,564	0.6970	0.8967	−0.06532	0.008093	0.8922
C17:2	341,915	332,180	0.7980	0.9351	0.04167	−0.003701	0.9506
C18:0, Stearic acid	318,734,394	332,710,839	0.2550	0.6394	−0.06191	−0.09662	0.1048
C18:1, Oleic acid	984,889,679	1,082,058,365	0.3170	0.6942	−0.13574	−0.09443	0.113
C18:2, Linoleic acid	538,584,601	607,601,438	0.1820	0.5581	−0.17395	−0.1037	0.0815
C18:4	846,890	1,031,088	0.1240	0.4632	−0.28392	−0.07859	0.1874
C19:0	1,160,760	1,203,104	0.5150	0.8113	−0.05169	−0.04858	0.4156
C19:1	3,089,727	3,347,807	0.2710	0.6534	−0.11574	−0.04832	0.418
C19:2	289,885	279,661	0.7170	0.9040	0.0518	0.03479	0.56
C20:3	7,012,135	9,263,184	<0.0001	0.0001	−0.40165	−0.2184	0.0002
C20:4, Arachidonic Acid	29,482,221	32,689,574	0.0929	0.3970	−0.14899	−0.141	0.0177
C20:7	26,077	41,257	0.2700	0.6529	−0.66186	−0.03019	0.613
C21:0	155,285	154,112	0.9370	0.9802	0.01094	−0.03349	0.5748
C21:1	142,277	141,117	0.9240	0.9752	0.01181	−0.03286	0.582
C21:3	207,492	313,653	0.0001	0.0013	−0.59612	−0.1867	0.0016
C21:6	822,345	1,339,443	0.4060	0.7518	−0.70382	−0.02205	0.7119
C22:2	552,154	596,054	0.4510	0.7773	−0.11037	−0.1474	0.0131
C22:3	281,365	356,169	0.0028	0.0313	−0.34012	−0.2054	0.0005
C23:1	474,394	582,737	0.0618	0.3098	−0.29676	−0.107	0.0723
C23:4	79,854	93,567	0.2300	0.6136	−0.22863	−0.04605	0.4403
C24:0	1,558,366	1,532,049	0.9010	0.9675	0.02457	−0.102	0.0869
C24:2	901,910	1,180,128	0.0007	0.0090	−0.38789	−0.3027	<0.0001
C24:4	121,348	169,090	0.0001	0.0010	−0.47864	−0.2798	<0.0001
C24:5	110,739	156,708	0.0043	0.0448	−0.50091	−0.2157	0.0003
C24:6	86,222	138,381	<0.0001	<0.0001	−0.68252	−0.2801	<0.0001
C25:0	671,084	995,834	0.0002	0.0037	−0.56941	−0.2458	<0.0001
C25:1	562,359	787,622	0.0047	0.0476	−0.48601	−0.2388	<0.0001
C25:2	167,295	295,182	<0.0001	<0.0001	−0.81921	−0.3181	<0.0001
C25:3	85,177	102,208	0.0933	0.3976	−0.26297	−0.08637	0.1473
C26:0	647,970	932,649	0.0008	0.0103	−0.52541	−0.2573	<0.0001
C26:1	570,410	816,052	0.0012	0.0150	−0.51666	−0.2603	<0.0001
C26:2	315,457	551,514	<0.0001	<0.0001	−0.80595	−0.3175	<0.0001
C26:3	197,298	354,612	<0.0001	<0.0001	−0.84587	−0.3508	<0.0001
C26:4	80,541	138,437	<0.0001	<0.0001	−0.78144	−0.3576	<0.0001
C26:5	49,512	72,036	<0.0001	<0.0001	−0.54093	−0.4672	<0.0001

## Discussion

The challenges associated with diagnosing and monitoring kidney AML/cysts in TSC patients highlight the need to develop simpler, more affordable, and less invasive screening methods. In our analysis of plasma samples from 283 AML/cyst-positive and -negative TSC patients, we identified octanoic acid and six additional chemical features that are significantly increased in AML/cyst-positive TSC patients as compared to AML/cyst-negative TSC patients.

A blood-based detection method could serve as a way for physicians to first screen patients prior to standard confirmatory imaging and biopsy techniques of kidney AML/cysts diagnosis. Future studies are needed to identify additional biomarkers that will enhance sensitivity by identifying the ∼20% of AML/cyst-positive patients who do not exhibit increased levels of any of the 7 candidate markers evaluated here (Figure 4). Of note, a similar screening approach has been developed to detect the TSC-associated condition lymphangioleiomyomatosis (LAM),^34^^,^^35^^,^^36^^,^^37^^,^^38^^,^^39^^,^^40^ with vascular endothelial growth factor-D (VEGF-D) being identified as a circulating biomarker that can be used toward LAM diagnosis.^39^^,^^40^ Future studies are required to determine whether our identified markers can determine stage or severity of disease and monitor drug effect (regression of AML/cysts).

The identified diagnostic markers may also provide insight into mechanisms by which kidney AML/cysts develop in TSC patients. We noted markedly elevated levels of octanoic acid and closely related metabolites such as putative 2-octenoic acid and hydroxy octanoic acid in a subset of AML/cyst-positive TSC patients (Figure 2). It is currently unclear why these metabolites are not uniformly elevated in AML/cyst-positive patients. These metabolites may be produced but then consumed by other organs or excreted more rapidly in some individuals, leading to less accumulation. Intriguingly, the strong negative correlation that we observed between these metabolites and several VLCFAs provides potential biological insights (Figure 5B; Table 3). VLCFAs are oxidized in peroxisomes, which are particularly abundant in the liver and kidney.^41^^,^^42^^,^^43^ During VLCFA oxidation in these organs, the peroxisome produces reactive oxygen species (ROS), which can cause *TSC1* and *TSC2* to associate with peroxin (PEX) proteins on the peroxisomal membrane. This in turn suppresses mTOR complex 1 (mTORC1) and triggers peroxisome degradation via autophagy.^44^^,^^45^ Peroxisome abundance is therefore negatively regulated by the TSC1/2-mTORC1 signaling-dependent “pexophagy”.^46^^,^^47^ However, when both alleles of *TSC1* or *TSC2* are mutated in AML/cysts, mTORC1 cannot be suppressed and peroxisomes can remain active and continue to oxidize VLCFAs. This could result in an increased production of octanoic acid, which may then be released into the bloodstream (Figure 6). This intriguing connection between *TSC* mutations and increased peroxisome activity may provide a biological explanation for the increased octanoic acid.

### Limitations of the study

There are some limitations in this study. First of all, our study involved one cohort of patients and thus another independent cohort will be required to validate our findings. Second, our proposed markers were identified solely based on the presence of kidney AML/cysts. However, TSC is a multi-organ disease and accordingly, it will be valuable to identify additional biomarkers and metabolic perturbations that can distinguish each organ’s tumors in TSC individuals. Finally, we were unable to determine the chemical identity of our markers except for octanoic acid. Therefore, further research is required to identify these chemical features, their source organs, and clinical implications.

## STAR★Methods

### Key resources table

**Table:** 

REAGENT or RESOURCE	SOURCE	IDENTIFIER
**Biological samples**		

Blood samples from TSC patients	TSC Alliance Biosample Repository	N/A
Blood samples from non-TSC patients	UC Irvine Experimental Tissue Resource Facility	N/A

**Chemicals, peptides, and recombinant proteins**		

Octanoic acid, analytical standard	Sigma-Aldrich	Cat# 21639

**Software and algorithms**		

R, version 4.1.3	R software	http://www.r-project.org
Analyse-it, version 6.15	Analyse-it Software, Ltd., Leeds, UK	https://analyse-it.com
BioRender	BioRender	https://www.biorender.com

### Resource availability

#### Lead contact

Further information and requests for resources and reagents should be directed to and will be fulfilled by the lead contact, Gina Lee (ginalee@uci.edu).

#### Materials availability

This study did not generate new unique reagents.

#### Data and code availability

• All data reported in this paper will be shared by the lead contact upon request.
• This paper does not report original code.
• Any additional information required to reanalyze the data reported in this paper is available from the lead contact upon request.

### Experimental model and study participant details

#### Sample collection and ethics

Blood samples were collected and provided by the TSC Alliance Biosample Repository (283 TSC patient samples) and UC Irvine Experimental Tissue Resource Facility (51 non-TSC patient samples). For a longitudinal study of TSC patients, blood was collected twice from the same patient with a mean interval of 843 days (range of 176-1642 days). Briefly, the blood collected in the ethylenediaminetetraacetic acid (EDTA)-coated vacutainer blood collection tube was centrifugated at 500g at 4°C for 10 min. The clear supernatant (plasma) was centrifugated again at 1,500g at 4°C for 10 min, and the supernatant was stored at -80°C until metabolomics analysis. All TSC subjects had a definite clinical diagnosis of TSC meeting the standard inclusion criteria.^17^ Clinical information was provided by the TSC Natural History Database and UC Irvine Health. The study was approved by the Institutional Review Board of Salus IRB Services (formerly known as Ethical & Independent Review Services) and UC Irvine. All samples and clinical information were collected in compliance with Salus IRB Services Protocol #15039 (TSC Alliance), UC Irvine IRB Protocol #2012-8716, and UC Irvine IRB Protocol #2021-6823 in accordance with the ethical standards of the responsible committee on human experimentation (institutional and national) and with the Helsinki Declaration of 1975, as revised in 2013.

### Method details

#### Measurements of metabolites using LC-MS

For aqueous metabolites extraction, serum (5 μL) was mixed with 150μl -20°C 40:40:20 methanol:acetonitrile:water (v:v:v, extraction solvent), vortexed and immediately centrifuged at 16,000g for 10 min at 4°C. The supernatant (70 μL) was loaded into individual LC-MS vials. Metabolites were analyzed by quadrupole-orbitrap mass spectrometer (Q-Exactive Plus Quadrupole-Orbitrap, Thermo Fisher) mass spectrometers coupled to hydrophilic interaction chromatography (HILIC) via electrospray ionization. LC separation was on an Xbridge BEH amide column (2.1 mm x 150 mm, 2.5 μm particle size, 130 Å pore size; Waters) at 25°C using a gradient of solvent A (5% acetonitrile in water with 20 mM ammonium acetate and 20 mM ammonium hydroxide) and solvent B (100% acetonitrile). The flow rate was 350 μL/min. The LC gradient was: 0 min, 75% B; 3 min, 75% B; 4 min, 50% B; 5 min, 10% B; 7 min, 10% B; 7.5 min, 75% B; 11 min, 75% B. Column temperature set to 25°C. Autosampler temperature was set at 4°C and the injection volume of the sample was 5 μL. MS analysis was acquired in negative ion mode with MS Full-scan mode from m/z 70 to 830 and 140,000 resolution. Data analysis was performed with Compound Discoverer and MAVEN software.

#### Gene expression analysis

The differentially expressed gene sets of the TSC renal angiomyolipoma (TSC-AML) and normal TSC kidney tissues were adopted from Martin et al., 2017.^32^ To assess the genes involved in peroxisome biogenesis and fatty acid beta-oxidation, the list of the genes under this functional category was retrieved from the QuickGO database (https://www.ebi.ac.uk/QuickGO/) and used to identify the gene expression changes in between the TSC AML and non-TSC kidneys.

### Quantification and statistical analysis

#### Statistical analysis

Selection criteria for determining chemical features of interest included: (1) **|**log_2_(fold change)**|** > 1; and (2) demonstration of a statistically significant difference between TSC patient with kidney AML or cysts and those with normal kidney using Student’s t-test with FDR correction. Mann-Whitney test was used to compare median of age between groups. Pearson’s correlation was used to evaluate the correlation between two variables. Comparison of categorical measures between independent groups was performed by Pearson’s chi-squared test. Student’s t-test was used to compare group means of peak area of chemical features and gene expression analysis. Statistical analyses were performed with R, version 4.1.3 (http://www.r-project.org) and Analyse-it (version 6.15, Analyse-it Software, Ltd., Leeds, UK). p < 0.05 was defined as statistical significance.
