# Supplementary material for: Circulating biomarkers of kidney angiomyolipoma and cysts in tuberous sclerosis complex patients
Source: iScience. 2024 Jun 13;27(7):110265. doi: 10.1016/j.isci.2024.110265 (PMC11255849; doi:10.1016/j.isci.2024.110265)
Supplement: Document S1. Figures S1–S6 and Tables S1–S7 [file mmc1.pdf]

iScience, Volume *27*

Supplemental information

Circulating biomarkers of kidney angiomyolipoma and cysts in tuberous sclerosis complex patients

Varvara I. Rubtsova, Yujin Chun, Joohwan Kim, Cuauhtemoc B. Ramirez, Sunhee Jung, Wonsuk Choi, Miranda E. Kelly, Miranda L. Lopez, Elizabeth Cassidy, Gabrielle Rushing, Dean J. Aguiar, Wei Ling Lau, Rebecca S. Ahdoot, Moyra Smith, Aimee L. Edinger, Sang-Guk Lee, Cholsoon Jang, and Gina Lee

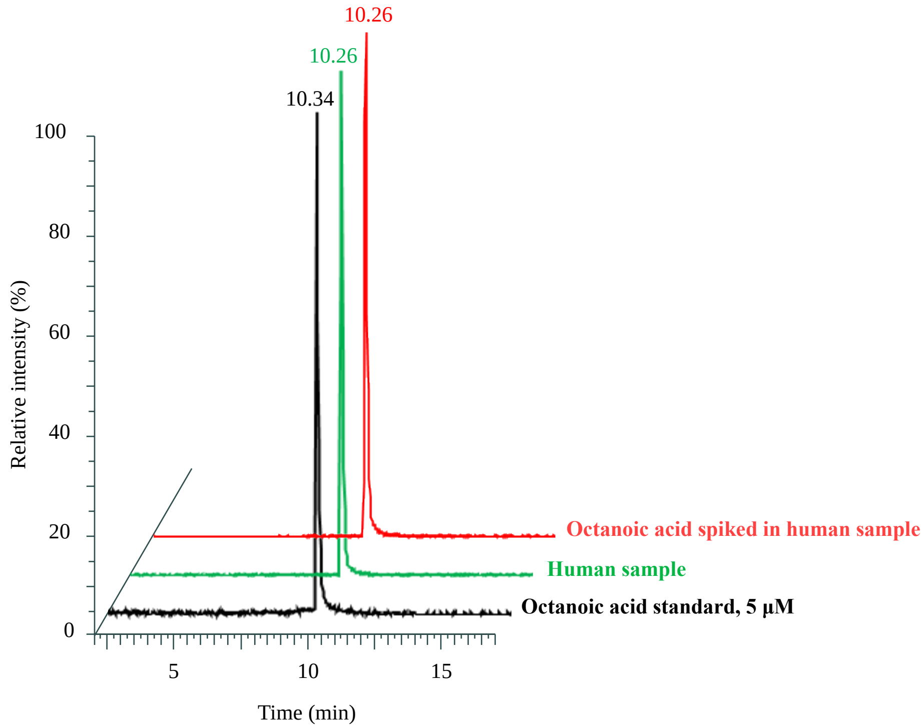

Figure S1. Identification of MW 144.11485 as octanoic acid, Related to Table 2. Octanoic acid dissolved in extraction solution shows one peak at 10.34 min (black) and octanoic acid spiked in the human blood sample shows one peak at 10.26 min (red), which matches with the peak in the human sample (green). This shift is likely due to the plasma matrix effect.

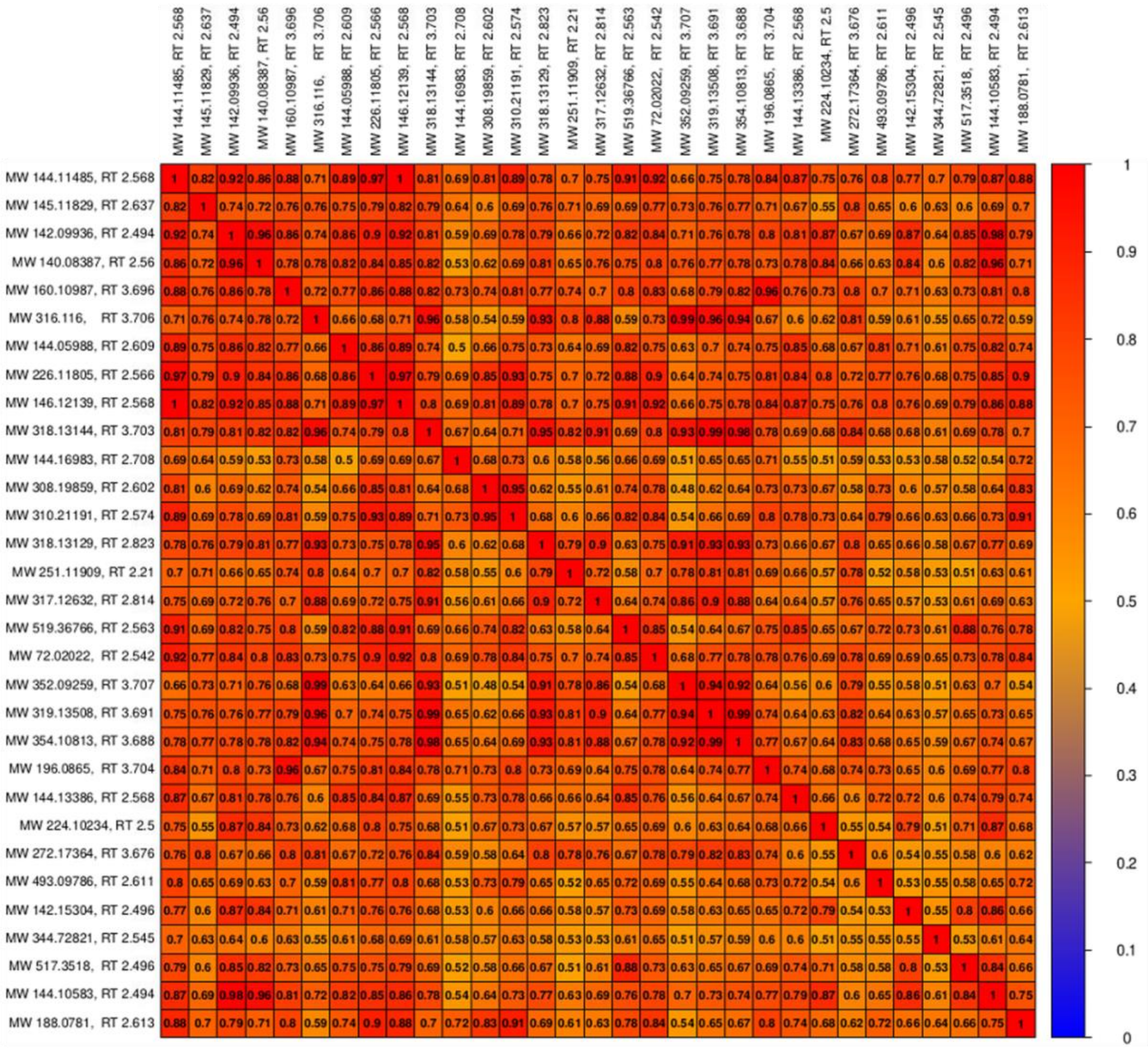

Figure S2. Correlation analysis (Pearson's r) between group 1 chemical features (N=31), Related to Figure 2.

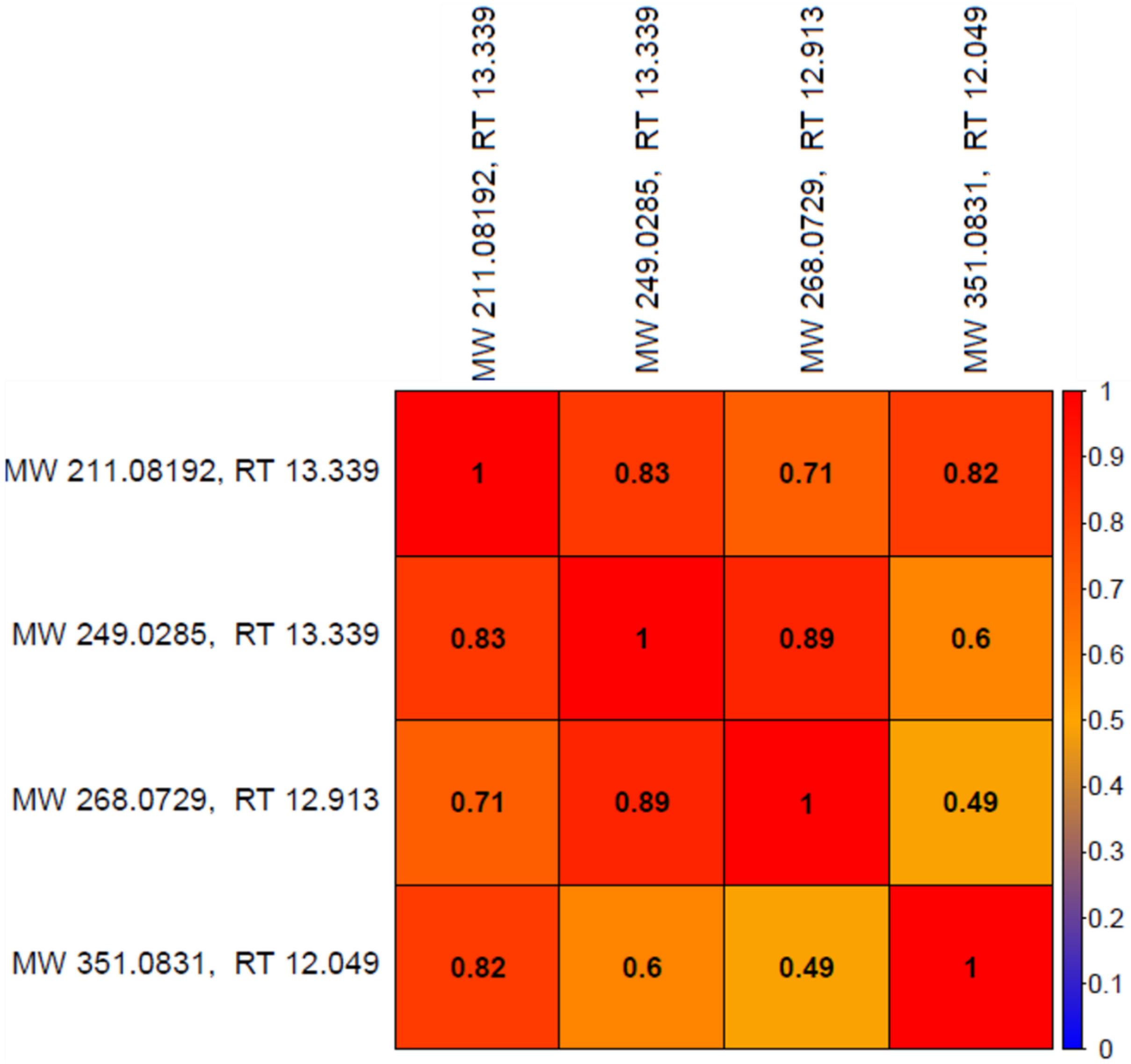

Figure S3. Correlation analysis (Pearson's r) between group 2 chemical features (N=4), Related to Table 2.

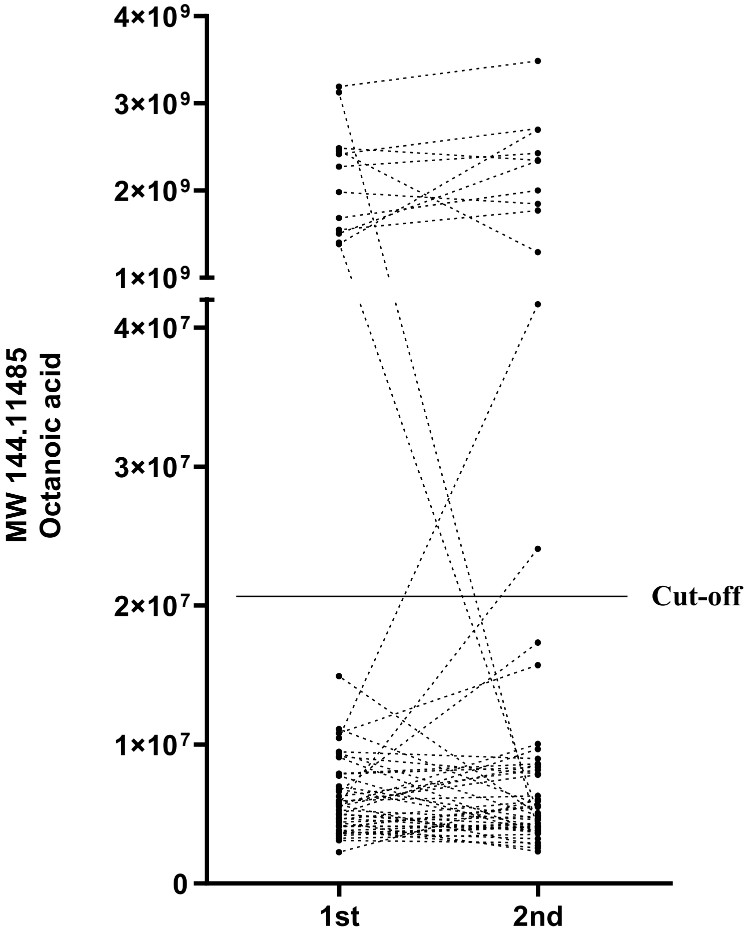

Figure S4. Changes in the peak areas of MW 144.11485 (octanoic acid) in the 55 patients who had follow-up measurements, Related to Figure 4.

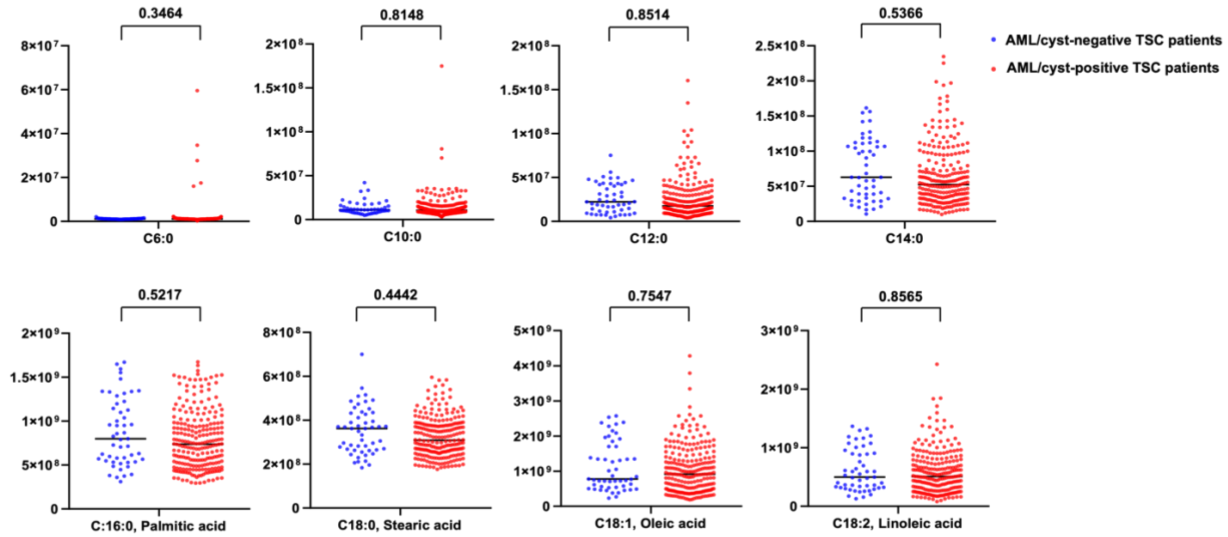

Figure S5. Comparison of medium and long chain free fatty acids between AML/cyst-negative vs. -positive TSC patients, Related to Table 3. There were no significant differences in medium or long-chain fatty acids except octanoic acid (C8:0). Numbers indicate p-values by Student's t-test.

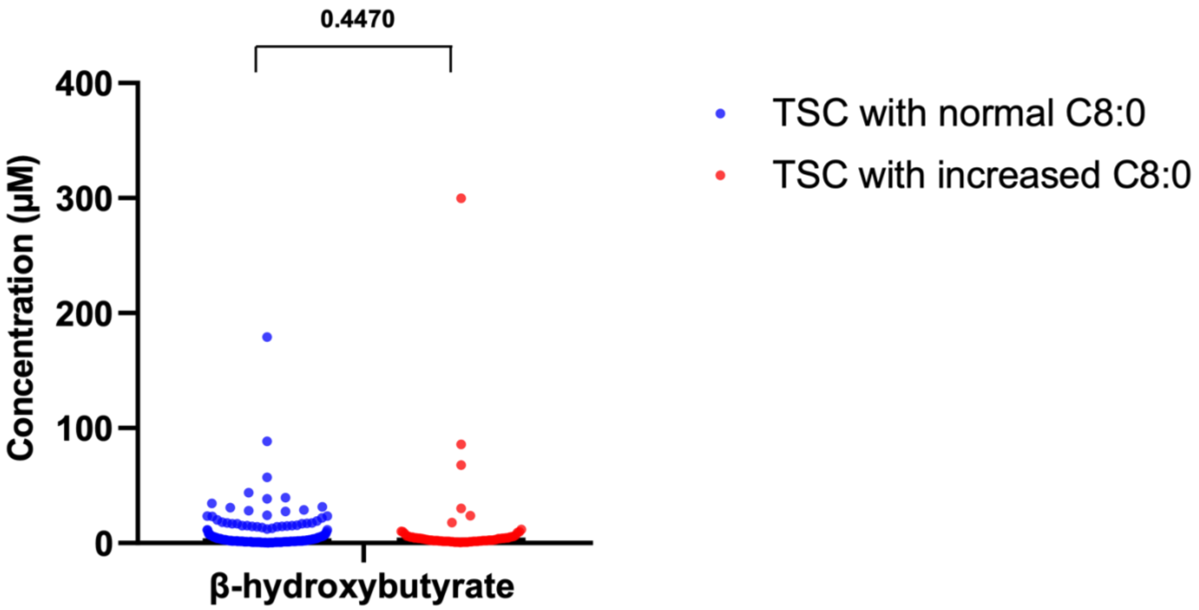

Figure S6. Comparison of β-hydroxybutyrate concentrations between the TSC patients with normal vs. increased octanoic acid, Related to Table 3. There is not a significant difference in β-hydroxybutyrate levels between the two groups. Number indicates a p-value by Student's t-test.

Table S1. Genetic test status and *PKD1* mutation result, Related to Table 1.

	Genetic test status of TSC patients (N=283)			
	Yes	No	Unknown	No answer
Number	218	43	17	5
PKD1 mutation, n (%)				
Yes	5 (2.3%)			
No	184 (84.4%)			
Unknown	28 (12.8%)			
No answer	1 (0.5%)			

Table S2. No effect of mTOR inhibitor use on octanoic acid level, Related to Table 1.

	Octanoic acid level, n (%)		
mTOR inhibitor use	Increase	Normal	Total
Yes	31 (26.5)	86 (73.5)	117 (100)
No	39 (23.5)	127 (76.5)	166 (100)

Pearson chi-squared test, p-value 0.5644

Table S3. Basic characteristics of non-TSC patients, Related to Figure 3.

	non-TSC patients (N = 51)
Age in years, median (range)	37 (15-60)
Gender, n (%)	
Women	26 (51.0)
Men	25 (49.0)
Health status, n (%)	
Presented for regular checkup	8 (15.7)
Minor pain or trauma in the upper or lower extremity	14 (27.5)
Cardiac problems	7 (13.7)
Generalized weakness	5 (9.8)
Minor skin disorders	3 (5.9)
Psychiatric problem	3 (5.9)
Migraine	2 (3.9)
Psychiatric problems	2 (3.9)
Anemia	1 (2.0)
Dyspnea	1 (2.0)
External hemorrhoids	1 (2.0)
Gallbladder sludge	1 (2.0)
Reproductive health problem	1 (2.0)
Sinusitis	1 (2.0)
Tinnitus	1 (2.0)

Table S4. Cut-off value of each chemical feature for increase (calculated as 90th percentile value from TSC patients with normal kidneys), Related to Figure 4.

ID	MW 144.11485	MW 211.08192	MW 315.24	MW 233.06623	MW 386.05722	MW 194.04241	MW 470.23385
TSC000028	18,082,391	6,092	145,760	711,628	12,887	14,671	20,681
TSC000035	6,128,824	6,410	136,066	167,294	8,367	42,737	23,504
TSC000051	5,301,161	6,492	163,753	30,434	8,650	30,803	19,929
TSC000076	5,152,849	6,419	185,788	40,790	8,475	24,506	16,275
TSC000114	4,455,966	5,705	498,201	543,187	10,199	22,684	41,929
TSC000116	5,485,988	6,018	856,915	145,114	8,799	24,681	51,261
TSC000128	1,883,415,288	6,164	252,166	522,875	67,496	197,107	48,782
TSC000150	10,956,924	7,898	184,879	10,465	8,908	25,245	24,838
TSC000177	2,197,371,380	11,745	358,227	405,766	78,174	53,216	12,342
TSC000195	3,894,417	6,409	277,212	63,428	8,621	23,655	9,536
TSC000249	6,498,153	6,350	189,363	54,254	10,444	92,066	16,257
TSC000290	5,409,412	6,845	340,751	105,315	10,290	15,330	12,746
TSC000316	5,108,975	6,039	494,088	122,814	9,685	23,130	31,283
TSC000358	5,627,365	6,026	120,839	1,140,056	7,260	23,334	40,292
TSC000412	4,148,741	5,508	134,590	42,530	7,100	22,711	28,897
TSC000794	3,251,038	6,404	147,075	706,277	7,134	37,577	7,767
TSC000795	3,793,424	5,408	160,365	57,647	6,714	34,549	16,189
TSC000966	5,560,112	5,293	403,509	433,932	8,590	91,877	53,929
TSC001057	4,385,061	56,013	118,013	97,415	8,586	29,679	25,094
TSC001401	4,694,799	6,059	168,406	77,180	9,452	25,012	66,423
TSC001649	3,970,852	5,945	434,163	220,727	8,246	29,365	15,947
TSC001694	5,098,272	6,397	179,436	15,330	8,442	20,945	20,878
TSC001771	12,679,255	4,972	160,610	69,048	8,036	22,995	11,274
TSC002170	5,299,314	5,712	209,320	313,664	9,540	44,422	44,473
TSC002948	3,640,008	6,276	302,460	12,349	12,220	28,650	14,108
TSC003461	3,643,122	57,491	140,088	451,253	8,157	31,660	42,617
TSC003465	5,718,045	6,411	159,597	85,416	36,580	28,697	12,225
TSC003742	4,009,490	5,987	155,976	276,781	8,456	24,484	13,173
TSC003786	3,791,777	12,932	149,572	654,803	10,756	29,578	21,637
TSC003790	5,574,177	6,379	507,677	1,324,278	8,120	25,722	16,991
TSC003793	4,924,946	5,934	305,229	565,925	9,185	23,105	53,588
TSC004052	1,414,148,302	10,468	312,274	419,977	47,563	135,852	12,027
TSC004056	5,689,042	6,140	197,830	1,049,717	11,531	45,050	41,631
TSC004062	7,124,625	6,306	161,011	554,851	8,170	26,408	26,332
TSC004164	11,802,408	9,551	175,614	186,709	24,590	88,365	14,031
TSC004172	3,609,201	6,174	162,497	67,634	15,665	23,395	12,580
TSC004246	8,736,359	6,966	237,906	9,909	7,823	23,118	14,063
TSC004263	4,133,206	6,310	147,190	99,291	8,120	24,187	21,354
TSC004272	21,375,265	6,230	566,831	20,938	8,906	26,700	27,769
TSC004287	4,856,716	6,425	346,346	184,971	8,423	23,050	38,759
TSC004391	10,435,182	6,036	137,323	16,023	15,917	48,792	12,447
TSC004531	3,652,335	5,582	366,033	199,111	7,428	23,972	12,121

TSC004661	5,056,109	5,955	651,048	14,982	7,571	41,396	55,653
TSC004781	5,779,764	61,384	346,971	357,052	11,976	50,051	15,117
TSC004858	7,353,998	182,297	149,930	292,644	7,837	40,184	11,896
TSC004952	2,055,523,138	5,279	125,597	686,116	68,278	36,207	19,812
TSC005000	4,390,982	5,268	179,341	598,813	6,611	24,700	28,463
TSC005013	4,020,846	5,769	191,008	156,171	7,664	25,019	43,321
TSC005015	7,003,419	6,164	127,977	520,509	52,736	27,440	30,245
TSC005030	3,916,915	5,248	387,357	67,945	7,100	20,904	14,093
TSC005040	14,398,176	4,906	329,199	33,118	6,386	27,634	47,367
90th Percentile cut-off	20,716,690	12,695	497,378	702,245	45,366	81,335	50,766

Table S5. Cut-off value of marker combination, Related to Figure 4.

Number of increased marker ≥	TP proportion (Sensitivity)	TN proportion (Specificity)	FP proportion	FN proportion	Odds ratio	Youden's index
**1**	**0.802**	**0.529**	**0.471**	**0.198**	**4.549**	**0.331**
2	0.448	0.824	0.176	0.552	3.792	0.272
3	0.319	0.961	0.039	0.681	11.475	0.280
4	0.211	1.000	0.000	0.789	+∞	0.211
5	0.103	1.000	0.000	0.897	+∞	0.103
6	0.034	1.000	0.000	0.966	+∞	0.034
7	0.009	1.000	0.000	0.991	+∞	0.009

Table S6. Changes in octanoic acid levels in the TSC patients with two measurements (average interval of 843 days, N=55), Related to Figure 4.

First sample result of octanoic acid	Follow up sample result of octanoic acid	Number of patients (Number of patients with normal kidney)
Normal	Normal	41 (7)
Normal	Increase	2 (0)
Increase	Increase	10 (0)
Increase	Normal	2 (0)

Table S7. Ketogenic diet status of TSC patients, Related to Figure 5.

	TSC patients (N = 283)
Ketogenic diet ongoing, n (%)	
Yes	2 (0.7)
No	219 (77.4)
Unknown	5 (1.8)
No answer	57 (20.1)
